# Belowground advantages in construction cost facilitate a cryptic plant invasion

**DOI:** 10.1093/aobpla/plu020

**Published:** 2014-04-30

**Authors:** Joshua S. Caplan, Christine N. Wheaton, Thomas J. Mozdzer

**Affiliations:** 1 Department of Biology, Bryn Mawr College, Bryn Mawr, PA, USA; 2 Smithsonian Environmental Research Center, Edgewater, MD, USA

**Keywords:** Carbon dioxide, common reed, construction cost, eutrophication, intraspecific, invasion ecology, *Phragmites*, plant functional traits, rhizomes, wetlands.

## Abstract

The energetic cost of plant organ construction is a functional trait that is useful for understanding carbon investment during growth (e.g. the resource acquisition vs. tissue longevity tradeoff), as well as in response to global change factors like elevated CO_2_ and N. Despite the enormous importance of roots and rhizomes in acquiring soil resources and responding to global change, construction costs have been studied almost exclusively in leaves. We sought to determine how construction costs of aboveground and belowground organs differed between native and introduced lineages of a geographically widely dispersed wetland plant species (*Phragmites australis*) under varying levels of CO_2_ and N. We grew plants under ambient and elevated atmospheric CO_2_, as well as under two levels of soil nitrogen. We determined construction costs for leaves, stems, rhizomes and roots, as well as for whole plants. Across all treatment conditions, the introduced lineage of *Phragmites* had a 4.3 % lower mean rhizome construction cost than the native. Whole-plant construction costs were also smaller for the introduced lineage, with the largest difference in sample means (3.3 %) occurring under ambient conditions. In having lower rhizome and plant-scale construction costs, the introduced lineage can recoup its investment in tissue construction more quickly, enabling it to generate additional biomass with the same energetic investment. Our results suggest that introduced *Phragmites* has had an advantageous tissue investment strategy under historic CO_2_ and N levels, which has facilitated key rhizome processes, such as clonal spread. We recommend that construction costs for multiple organ types be included in future studies of plant carbon economy, especially those investigating global change.

## Abstract (precis)

Energetic costs of tissue construction were compared in two subspecies of *Phragmites australis*, the common reed – namely the primary native and introduced lineages in North America. Caplan *et al*. report that the introduced lineage has lower construction costs than the native under all environmental conditions assessed, driven mainly by its lower cost rhizomes. These results highlight the fact that belowground energetics, which are seldom investigated, can influence the performance advantages that drive many plant invasions. The authors also demonstrate that tissue construction costs in organs not typically assessed can shift with global change, suggesting that they may have increasingly important implications into the future.

## Introduction

The energetic requirement of plant tissue biosynthesis, or construction cost (CC), has proven to be a valuable functional trait in investigations of the carbon economy of plants. Research on leaf CC and associated traits has yielded insights into the strategies used by plants for carbon acquisition (investment in leaf longevity, payback time for the investment, light harvesting area, etc.) and has thereby helped to explain patterns in growth at the individual and population levels (Wright *et al.* 2004; Poorter and Bongers 2006). For instance, a number of studies on invasive species have found lower leaf CCs, higher specific leaf areas (SLAs) and more rapid growth rates relative to co-occurring non-invasive species across life forms (Baruch and Goldstein 1999; Nagel and Griffin 2001; Deng *et al.* 2004; Feng *et al.* 2008; Osunkoya *et al.* 2010; Shen *et al.* 2011). Research on leaf CC has also identified ways in which plants will adjust leaf structure and function as changes in global climate intensify. In prior studies, most species decreased leaf CCs in response to elevated CO_2_ (Poorter *et al.* 1997; Lei *et al.* 2012), while leaf CC rose in response to higher nitrogen availability (Griffin *et al.* 1993).

Although functional trait studies that have included CC have almost exclusively used it to gain insight into the carbon economy of leaves, CC is not a trait specific to leaves. The few studies that have addressed CCs of roots, rhizomes or other organs have shown that high investment in one organ does not necessarily correspond to high investment in another (Wullschleger *et al.* 1997; Nagel *et al.* 2005; Osunkoya *et al.* 2008). Given that changes in biomass allocation and tissue composition have been observed in many species following CO_2_ and nitrogen manipulation (Poorter *et al.* 1997, 2012; Curtis and Wang 1998; Booker *et al.* 2000; Booker and Maier 2001; Kraus *et al.* 2004), changes in the CCs of organs other than leaves are probably common as well. Research explicitly investigating the CC of belowground organs in response to additions of CO_2_ and inorganic nitrogen would be especially useful in understanding how global change will affect the trajectory of plant populations as resource regimes shift.

*Phragmites australis*, or common reed (hereafter *Phragmites*), is well suited for an investigation of how plants may adjust tissue construction in response to global change. *Phragmites* has a cosmopolitan distribution, with dozens of genetic lineages in the species (Saltonstall 2002; Lambertini *et al.* 2012). It is therefore possible to tightly constrain phylogeny while comparing CCs between lineages that co-occur in natural ecosystems. The most well-studied case is that of two lineages that occur in tidal wetlands along the Atlantic coast of North America. One lineage was introduced from Eurasia to North America in the mid-1800s (haplotype M; *P. australis* subsp. *australis*; hereafter ‘introduced *Phragmites*’) (Saltonstall 2002). It has invaded wetlands across the Atlantic coast of North America, dramatically changing both ecosystem structure and function (Marks *et al.* 1994; Chambers *et al.* 1999; Kettenring *et al.* 2012). The other lineage present is a haplotype native to the region (haplotype F; *P. australis* subsp. *americanus*; hereafter ‘native *Phragmites*’) (Saltonstall 2002).

Strong differences in physiology, growth (aboveground and belowground) and abundance have been observed between native and introduced *Phragmites* (Saltonstall 2007; Saltonstall and Stevenson 2007; Park and Blossey 2008; Mozdzer and Zieman 2010; Mozdzer *et al.* 2010, 2013). Further, differences in growth rate between the lineages are known to become exacerbated in response to eutrophication and elevated atmospheric CO_2_ (Saltonstall and Stevenson 2007; Holdredge *et al.* 2010; Mozdzer and Megonigal 2012; Tulbure *et al.* 2012; Mozdzer *et al.* 2013). Eutrophication is probably one of the primary drivers of the introduced lineage spreading rapidly in many wetland ecosystems. For instance, its abundance is correlated with shoreline development (King *et al.* 2007), a process that combines elevated nutrient availability, habitat modification and diminished salinity (Silliman and Bertness 2004). Introduced *Phragmites* is able to achieve particularly high rates of seedling establishment and growth in such environments, and also experiences higher rates of outcrossing (rather than self-pollination; McCormick *et al.* 2010). Because outcrossing is associated with greater seedling production, the availability of eutrophied environments is hypothesized to accelerate invasion dramatically (McCormick *et al.* 2010; Hazelton *et al.* 2014). In the context of rising atmospheric CO_2_ and intensifying anthropogenic disturbance in wetland systems, information on how introduced *Phragmites* invests in tissue construction, and how it adjusts this investment in response to the environment, could be highly relevant in understanding the ecological processes driving the invasion, as well as in formulating strategies to manage it.

We sought to determine how CCs of plant organs in introduced and native *Phragmites* lineages would vary in response to alterations to CO_2_, nitrogen (N) and the combination of these factors. We measured leaf, stem, rhizome and root CCs in greenhouse-grown plants, and compared organ-specific and whole-plant CCs with other functional traits related to growth and morphology. In keeping with prior observations of leaves in invasive species, we hypothesized that CCs of all organ types, as well as whole plants, would be lower for introduced vs. native *Phragmites*. Further, we hypothesized that the difference in CCs between lineages would increase when plants grew under levels of CO_2_ or inorganic N expected in the coming century (Hopkinson and Giblin 2008; Meinshausen *et al.* 2011), with the greatest difference being in plants that experienced higher CO_2_ and N simultaneously.

## Methods

*Phragmites australis* plant material was originally collected from marshes on the Delmarva Peninsula, USA (38.5°N, 75.5°W); populations of native and introduced *Phragmites* were sampled from stands that were located within 50 km of one another. Samples were genetically confirmed to belong to haplotypes F and M, which correspond to North American Atlantic coast native and Eurasian introduced lineages, respectively. Clones from this material were subsequently grown in a common garden at the University of Rhode Island, where they experienced identical abiotic conditions for 3 years (2006–09). We therefore attribute any differences in functional trait expressions between lineages from this experiment strictly to the genetic source. Plants for the experiment described herein were propagated from rhizome fragments at the Smithsonian Environmental Research Center in Edgewater, MD, USA in 2009, where the experiment also took place. Rhizome fragments contained 3–5 intact internodes, which was equivalent to 1.29 ± 0.07 and 1.10 ± 0.70 g (mean ± SE) dry mass for native and introduced lineages, respectively. Rhizomes were planted individually in plastic pots (15 L; 24 × 24 × 33 cm) that contained reed-sedge peat (Baccto, Houston, TX, USA) on 11–12 June 2009.

The experiment had a three-way factorial design, which included two levels of atmospheric CO_2_, two levels of soil N and the two *Phragmites* lineages. Plants from each lineage were randomly distributed among six transparent chambers, in which CO_2_ was either not added or elevated to ∼330 ppm above ambient air (Mozdzer and Megonigal 2012). This is a conservative estimate of rise in global mean CO_2_ concentration by the latter part of the 21st century (Meinshausen *et al.* 2011). Plants were placed in chambers when new growth became visible at the soil surface; the first plant emerged on 19 June 2009. Within each chamber, half of the plants from each lineage received supplemental N at a rate equivalent to 25 g m^−2^ year^−1^, while the remaining half were unfertilized. The higher N level is typical of those seen in eutrophied tidal marsh ecosystems (Hopkinson and Giblin 2008). Nitrogen was delivered bi-weekly via a solution of NH_4_Cl. A sufficient quantity of tapwater to maintain at least 3 cm of standing water was added to each pot daily. To allow for water movement through the potting medium, four macropores were inserted vertically using PVC tubing (1.25 cm i.d.).

Plants were destructively harvested after ∼2 months of exposure to treatment conditions (20–27 August 2009). Material from each individual (*N* = 52) was carefully separated into leaf, stem (culm plus leaf sheath), rhizome and root categories. All plant material was oven dried at 60 °C to constant mass, weighed and finely ground. Samples of ground tissue were analysed at the University of Virginia for elemental carbon and nitrogen content (Carlo Erba Instruments, NA2500, Milan, Italy). Tissue mineral content was determined via loss-on-ignition using a separate set of samples; ∼0.5 g of each sample was ashed in a muffle furnace for 6 h at 550 °C.

Organ-specific CCs were determined using a method based on the production value of dry matter. Construction cost is defined specifically as the mass of glucose required to synthesize a given mass of plant tissue, but can be determined from the carbon (C_dm_) and ash (Ash_dm_) content of dried organic material as follows (Vertregt and Penning de Vries 1987):

$$\hbox{CC} = {5.39\hbox{C}_{{\rm dm}} + 0.80\hbox{Ash}_{{\rm dm}} -1191\over 1000}.$$

While estimates of CC are more complicated when the N source available to plants includes NO_3_ (Vertregt and Penning de Vries 1987; Poorter *et al.* 1997), NH_4_ was the sole N source in this experiment. Further, very little of the NH_4_ could have oxidized given that soils were constantly inundated; measurements of redox potential confirmed that soils were predominantly anaerobic (Mozdzer and Megonigal 2013). After calculating CCs for each organ type (CC_org_, where org is alternately leaf, stem, rhizome or root), we determined the contribution of organ-specific CCs (Contrib_org_) to plant-scale CCs (CC_plant_) by weighting CC_org_ by the corresponding mass fraction (MF_org_; organ mass per plant mass) and summing the contributions:

$$\eqalign{ {\hbox{Contrib}_{\rm org}} = {\hbox{CC}_{\rm org}} \times {\hbox{MF}_{\rm org},} \cr {\hbox{CC}_{\rm plant}} = {\hbox{Contrib}_{\rm leaf}} + {\hbox{Contrib}_{\rm stem}} + {\hbox{Contrib}_{\rm root}} \cr \quad + {\hbox{Contrib}_{\rm rhizome}}.} $$

Additional functional traits were measured for each plant. Relative growth rate (RGR) was based on the accumulation of dry biomass between planting (*M*_p_) and harvest *(M*_h_):

$$\hbox{RGR} = {({\ln ({M_{\rm h} } )-\ln ({M_{\rm p} } )} )\over t},$$

where *t* is the number of days between emergence and harvest (mean ± SE: 58 ± 1 days). Masses at the time of planting (*M*_p_) were determined from the fresh masses of rhizome fragments used to propagate plants; the water content of rhizome fragments that were not used in the study was used to estimate dry masses. Dry masses at harvest (*M*_h_) were sums of leaf, stem, rhizome and root masses. Stem heights and diameters were calculated as the mean of all stems in individual pots, with diameters measured at the soil surface. Stem density was a count of the number of stems per pot. Specific leaf area was calculated as the ratio of the area of the leaf blade to the dry mass of the third-most apical, fully developed leaf. Leaf blade areas were measured with an LI-3000 leaf scanner (LI-COR Biosciences, Lincoln, NE, USA). Additional procedural details are provided elsewhere (Mozdzer and Megonigal 2012, 2013).

Differences among experimental factors (CO_2_, N and lineage) with respect to CCs (plant scale and organ specific) were evaluated with ANOVA-type linear models in R version 3.0.2. Transformations to response variables (square root or natural log) were made if residuals were not normal and homoscedastic. Models initially contained terms for all main effects and interactions; when *F* statistics for individual terms (especially interactions) or the model itself were non-significant (using *α* = 0.05), simpler models were sought by sequentially removing non-significant terms. If an interaction term was significant, all lower-order terms were retained regardless of significance. Tukey's honestly significant difference (HSD) tests were used to evaluate pairwise differences among means based on terms in the final models. We assessed the correlation (Pearson coefficient, *ρ*) between CCs and other functional traits using mean values for each lineage within each combination of treatments. Variables for which *ρ* > 0.7 were considered strongly correlated, as this level of correlation corresponds to ∼50 % of the variation in CCs being explained by the functional traits in question (Sokal and Rohlf 1995).

## Results

**Figure 1.**
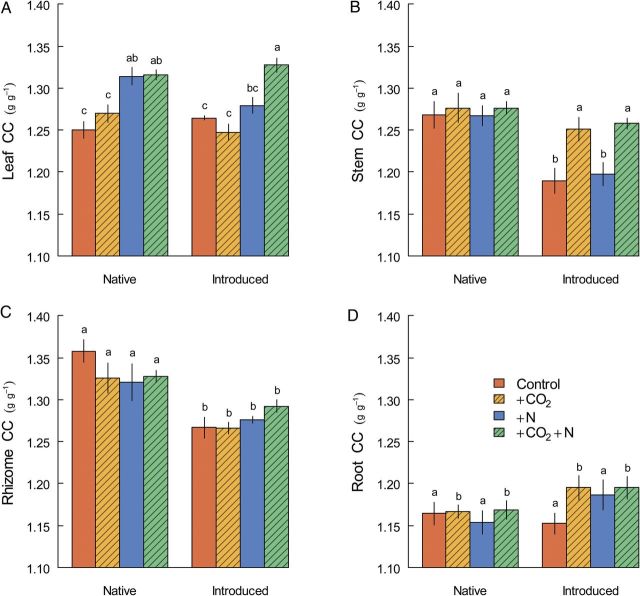
Organ-specific construction costs (CCs) for *Phragmites* lineages native to the North American North Atlantic coast (‘Native’) and introduced from Eurasia (‘Introduced’). Bar heights represent mean (±SE) CC for all plants grown in a combination of CO_2_ and N fertilization treatments. Within each panel, lowercase letters above bars differ when Tukey's HSD tests for the best-fitting model identified statistically significant differences in means. Units are grams of glucose required per gram of biomass produced.

The influence of lineage and environmental manipulations on CCs was strongly organ specific. Aboveground, leaf CCs were influenced by both N and CO_2_ treatment, but the magnitude of these effects depended on lineage (Fig. 1A, Table 1). Specifically, N fertilization induced an increase in leaf CC for native *Phragmites*, but this effect was independent of the CO_2_ level. In contrast, introduced *Phragmites* only increased its leaf CC with fertilization if CO_2_ was elevated as well. When averaging across environmental treatments, leaf CCs were similar for the two lineages. Unlike leaves, the CC of stems was unaffected by N fertilization, but did differ by lineage and CO_2_ status (Fig. 1B, Table 1). Specifically, under ambient CO_2_ conditions, introduced *Phragmites* generated stems that had 5.8 % lower CCs than did native *Phragmites*.

**Table 1.** Statistical results for the linear models best describing CCs as a function of *Phragmites* lineage and environmental treatments. Values in the top row for each model correspond to *F*-tests of each model as a whole, while the remaining values correspond to *F*-tests of individual terms.

Model	d.f.	*F*	*P*
Plant CC	4	16.07	<0.001
Lineage	1	31.99	<0.001
CO_2_	1	13.05	<0.001
N	1	14.30	<0.001
Lineage × CO_2_	1	4.96	0.031
Residuals	46		
Leaf CC	7	13.85	<0.001
Lineage	1	1.68	0.20
CO_2_	1	6.98	0.011
N	1	74.43	<0.001
Lineage × CO_2_	1	0.012	0.91
Lineage × N	1	0.059	0.81
CO_2_ × N	1	2.67	0.11
Lineage × CO_2_ × N	1	11.11	0.002
Residuals	43		
Stem CC	3	16.88	<0.001
Lineage	1	27.04	<0.001
CO_2_	1	14.65	<0.001
Lineage × CO_2_	1	8.95	0.004
Residuals	47		
Rhizome CC	1	36.35	<0.001
Lineage	1	36.35	<0.001
Residuals	49		
Root CC	2	3.91	0.027
Lineage	1	3.57	0.065
CO_2_	1	4.24	0.045
Residuals	48

The largest difference in CC between lineages was seen belowground, specifically in rhizomes. Rhizome CCs were 4.3 % lower for introduced *Phragmites* than for the native, and this difference was not significantly influenced by environmental treatments (Fig. 1C, Table 1). Root CCs were notably lower than they were for any other organ (Fig. 1D). Elevated CO_2_ induced slight increases in root CC for both lineages, while N fertilization had no measurable effect (Table 1). Although there was a trend towards higher root CC for introduced *Phragmites* compared with the native, this effect was not significant.

**Figure 2.**
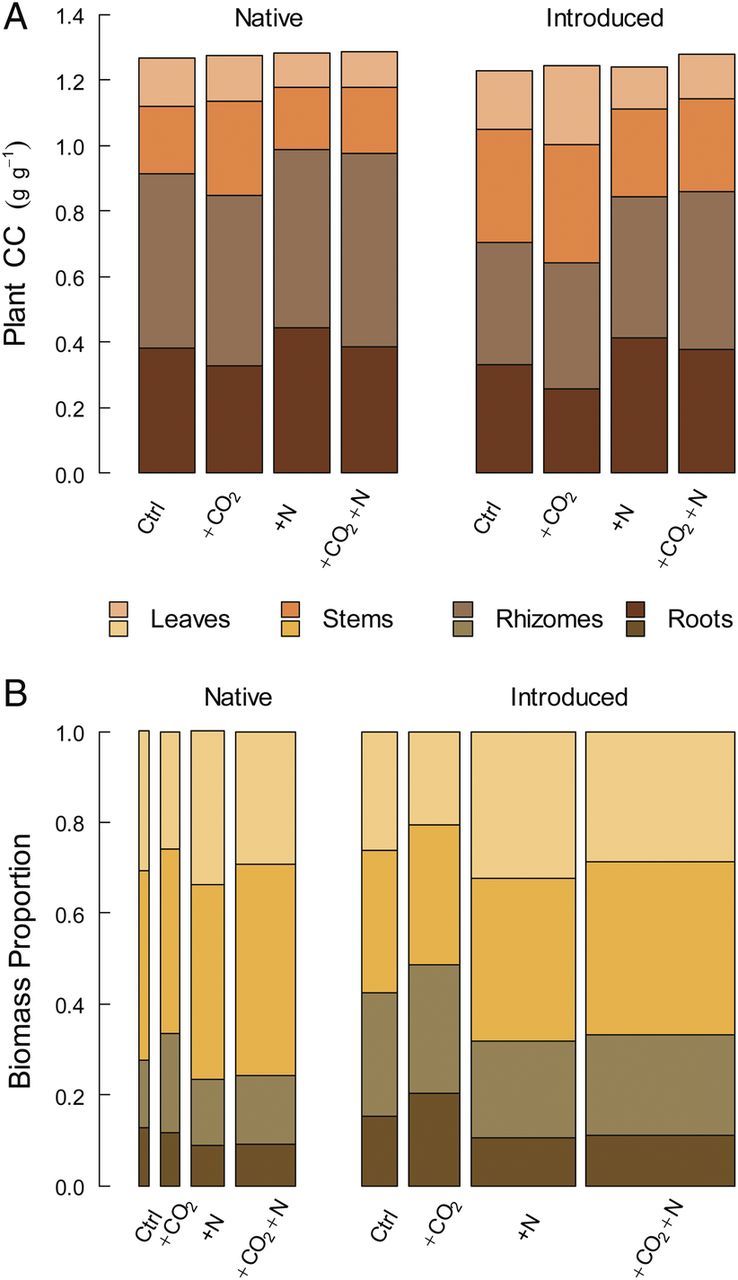
Partitioning of (A) plant-level CCs and (B) plant biomass by organ type for native and introduced *Phragmites* under each of the environmental treatment combinations evaluated in this experiment. Mean values for the replicate individuals within each treatment are shown. The widths of bars in (B) are scaled to the total biomass produced across treatments.

At the level of the whole plant, CCs differed by lineage and by environmental conditions. Introduced *Phragmites* had a lower mean CC than did the native (Table 1); the magnitude of this effect ranged from 0.6 to 3.3 % depending on the CO_2_ and N treatment levels, and was 2.3 % for all treatments pooled (Fig. 2A). Native and introduced *Phragmites* also differed markedly in the size of contribution that each type of organ made to whole-plant CCs. Under all environmental conditions, introduced *Phragmites* had smaller rhizome and root contributions, but larger stem and leaf contributions compared with the native. Relative to unfertilized conditions, elevated nitrogen raised the contribution of belowground organs to whole-plant CCs in both lineages. These differences in aboveground vs. belowground contributions were driven by CCs and not biomass distributions, as organ mass fractions were higher belowground for the introduced lineage and under fertilized conditions (Fig. 2B). The addition of CO_2_ also raised the contribution of aboveground organs to plant CCs over ambient conditions.

Across environmental treatments, higher leaf, rhizome and whole-plant CCs corresponded to introduced *Phragmites* plants growing faster, more densely and larger (taller, more massive and having wider stems; Table 2, Fig. S1 **[see Supporting Information]**). Correlation coefficients were consistently strongest for rhizome CCs in all of these relationships. Native *Phragmites* likewise grew more rapidly, more densely and larger as plant and leaf CCs increased. In contrast to the introduced lineage, rhizome CC in the native was oppositely, and generally more weakly, correlated to these and other traits than were leaf and whole-plant costs (Table 2). While SLA was negatively correlated with plant and stem CCs for the introduced lineage, it was positively correlated with plant and leaf CCs in the native (Table 2). Finally, for introduced *Phragmites*, leaf and rhizome CCs were strongly and positively associated with the N : C ratio of belowground organs, but only weakly associated with the N : C ratio of aboveground organs (*ρ* ≈ 0.6). The native lineage had strong positive associations between leaf and plant CCs and the N : C ratio of all organ types (Table 2).

**Table 2.** Correlation coefficients between whole-plant or organ-specific CCs and other functional traits. The underlying data were the means for each combination of environmental treatments (Control, +CO_2_, +N and +CO_2_+N) within each lineage. Pearson coefficients >0.7 are shown; all coefficients and plots of the underlying data appear in Fig. S1 **[see Supporting Information]**. RGR, aboveground relative growth rate; Height, mean height of stems; Stem diam., mean basal diameter of stems; Biomass, total plant biomass; Density, number of stems per pot; SLA, specific leaf area; N : C, nitrogen to carbon ratio in tissue. All traits were measured at harvest except RGR.

	Plant CC	Leaf CC	Stem CC	Rhizome CC	Root CC
Introduced *Phragmites*					
RGR	0.86	0.87	–	0.94	–
Height	0.93	0.92	–	0.98	–
Stem diam.	0.88	0.97	–	1.00	–
Biomass	0.86	0.91	–	0.97	–
Density	0.79	0.84	–	0.91	–
SLA	−0.87	–	−0.98	–	–
N : C_lf_	–	–	–	–	–
N : C_stem_	–	–	–	–	–
N : C_rhiz_	–	0.89	–	0.91	–
N : C_root_	–	0.78	–	0.79	–
Native *Phragmites*					
RGR	0.99	0.96	–	−0.75	–
Height	0.73	–	0.88	−0.73	–
Stem diam.	0.92	0.80	–	−0.72	–
Biomass	0.94	0.89	–	–	–
Density	0.93	0.94	–	–	–
SLA	0.90	0.86	–	−0.98	–
N : C_lf_	0.76	0.88	–	–	–
N : C_stem_	0.87	0.93	–	–	–
N : C_rhiz_	0.88	0.93	–	–	–
N : C_root_	0.83	0.94	–	–	–

## Discussion

### Rhizome construction costs

This study demonstrates that CCs for organs not typically measured (rhizomes, roots and stems) can reveal patterns of plant adaptation well beyond those that can be gleaned from leaf CC alone. Most strikingly, our results identified key advantages in rhizome investment for introduced *Phragmites* over the native lineage that likely contribute to its invasion as a perennial, clonal grass. By maintaining lower CCs under all combinations of CO_2_ and nitrogen, introduced *Phragmites* can recoup its investment in tissue construction more quickly (Poorter *et al.* 2006), enabling it to generate additional rhizome biomass and potentially other organs as well. Prior research supports this explanation; under multiple CO_2_ and N conditions and in multiple studies, introduced *Phragmites* had greater absolute rhizome mass, higher rhizome mass fractions, higher ramet densities and greater leaf areas compared with the native (League *et al.* 2006; Holdredge *et al.* 2010; Mozdzer and Megonigal 2012).

We suggest that the lower rhizome CC of introduced *Phragmites* ultimately contributes to advantages in belowground dynamics that are known to facilitate its invasion in North American tidal marshes. More specifically, we suggest that lower rhizome CC and shorter payback times allow introduced *Phragmites* to build more extensive rhizome systems (e.g. greater biomass, as seen in this study, as well as greater total length) than it would if CCs were higher. Low rhizome CC may also yield thicker rhizomes (i.e. higher masses per unit length, which could come from greater diameters, as seen by Holdredge *et al.* (2010), and/or from more dense rhizome tissue). Given that clonal expansion occurs by stems emerging from laterally extending rhizomes (Amsberry *et al.* 2000), the favourable carbon economics of rhizome generation may facilitate higher rates of ramet production, spatially and/or temporally, as reported for introduced vs. native lineages previously (Vasquez *et al.* 2005; League *et al.* 2006; Holdredge *et al.* 2010; Mozdzer and Megonigal 2012). By building and maintaining a network of stems that are connected by rhizomes, *Phragmites* clones can draw oxygen into belowground organs, aiding respiration and nutrient uptake (Brix *et al.* 1992; Vretare and Weisner 2000; Tulbure *et al.* 2012). Introduced *Phragmites* is able to induce 4× the rate of airflow per unit of pressure differential and stand area than native *Phragmites* (Tulbure *et al.* 2012). This efficiency is due, in part, to the higher stem densities of its clones (Rolletschek *et al.* 1999; Tulbure *et al.* 2012). In addition, the ability of introduced *Phragmites* to tolerate substantially higher salinity than native *Phragmites* contributes to its ability to invade habitats that the native lineage is unable to colonize (Vasquez *et al.* 2005). Tolerance to relatively high salinity (≤0.40 M NaCl) has been attributed to larger rhizome sizes, greater rhizosphere oxygenation and more rapid clonal growth by the introduced lineage (Vretare and Weisner 2000; Bart and Hartman 2003; Vasquez *et al.* 2005).

The advantages that introduced *Phragmites* exhibits in connection with low rhizome CC and short payback times, compared with native *Phragmites* and likely other species, are magnified by its rapid photosynthetic rates. The photosynthetic capacity (*A*_max_) of the introduced lineage has been measured as being 12–80 % higher than that of the native (Hansen *et al.* 2007; Mozdzer and Zieman 2010; Mozdzer *et al.* 2013). This translated into the introduced lineage producing more than twice as much rhizome biomass as native *Phragmites* in both field and greenhouse settings (Holdredge *et al.* 2010; Mozdzer and Zieman 2010; Mozdzer and Megonigal 2012). As past studies of photosynthetic traits did not manipulate CO_2_ or N, ecophysiological data collected under predicted future conditions would be extremely valuable in assessing the carbon economy of *Phragmites* as global change intensifies.

Our results also suggest that introduced *Phragmites* may avoid a tradeoff in photosynthate allocation between rhizomes and leaves. This is supported by the fact that rhizome CCs were positively correlated with leaf CCs and metrics of plant size in the introduced lineage, but negatively correlated in the native lineage. An ability to make a large investment in rhizomes may lead to greater root production and nutrient acquisition rates for introduced *Phragmites* (Holdredge *et al.* 2010), positively feeding back to growth and tissue quality both aboveground and below. We suspect that the native lineage is not sufficiently productive to support the initial investment in rhizome biomass needed to make such feedback possible.

There are several possible changes in rhizome tissue composition that could contribute to the observed differences in CCs between lineages. One possibility is that introduced *Phragmites* invests in a lower proportion of energetically expensive compounds like lignins, proteins and phenolics in rhizomes. Because they have larger diameters (Holdredge *et al.* 2010), rhizomes of introduced *Phragmites* may require less structural support via lignification. As described above, synthesis of fewer expensive compounds in rhizome tissue would lead to lower longevity, but a faster payback time, and a more rapid growth rate (Poorter *et al.* 2006). It is also possible that introduced *Phragmites* incorporates a greater proportion of inexpensive compounds than the native, such as non-structural carbohydrates or organic acids (Poorter *et al.* 2006). For instance, it may synthesize a larger surplus of starch via photosynthesis, much of which it may allocate belowground for immediate growth or storage (Granéli *et al.* 1992). Through this mechanism as well, introduced *Phragmites* would be able to achieve a rapid return on the energetic investment in rhizome tissue, facilitating its further growth.

### Response to global change factors

In direct contrast to prior studies (Poorter *et al.* 1997; Wullschleger *et al.* 1997; Nagel *et al.* 2004, 2005), all statistically separable comparisons of mean CCs for ambient vs. elevated CO_2_, as well as most of the non-significant comparisons, involved increases in CCs. However, the vast majority of past studies focused specifically on CCs of leaves. As seen in other studies that manipulated CO_2_ (Poorter *et al.* 2012; Langley *et al.* 2013; Madhu and Hatfield 2013), belowground production increased under elevated CO_2_ for both *Phragmites* lineages. The concomitant rise in root CCs may have been due to shifts in root morphology or architecture, such as larger diameters, higher tissue density or more frequent branching (Madhu and Hatfield 2013). Such shifts allow for increased nutrient uptake, soil penetration ability and resistance to pathogens and herbivores, but require increased synthesis of energetically expensive compounds like lignin and suberin (Vance *et al.* 1980; Soukup *et al.* 2002; Baxter *et al.* 2009). Consistent with this possibility, prior studies have found higher lignin concentrations in fine roots under elevated CO_2_ (Booker *et al.* 2000; George *et al.* 2003). The strong correlation of stem CCs with plant height in native *Phragmites* raises the possibility that stems were also more lignified under elevated CO_2_. Introduced *Phragmites* likewise exhibited a positive correlation between these factors, though it was only moderate in strength (*ρ* = 0.58).

Despite the literature's enormous emphasis on leaf CCs, we found no differences in leaf CC between lineages. While both lineages adjusted leaf CC in response to nitrogen addition, the magnitude of response was similar. Higher N availability probably corresponded to a greater investment in rubisco and other compounds associated with photosynthetic capacity (Griffin *et al.* 1993; Poorter and Bongers 2006). Other studies have also found negative correlations between leaf CC and SLA (e.g. Feng *et al.* 2008), whereas we found a positive correlation. We attribute this discrepancy to the fact that most other studies describe variation among species grown under similar environmental conditions, while our analysis portrays phenotypic plasticity in leaf construction to strongly varying environmental conditions. If we had only investigated leaf CCs for these *Phragmites* lineages, we would have overlooked key differences belowground, and determined little about the carbon economy or differences in invasiveness between lineages.

Our findings on whole-plant CCs suggest that an ability to generate biomass with a relatively short return time on the energetic investment has facilitated introduced *Phragmites* colonizing wetlands in North America over the past century (Saltonstall 2002). Modest differences in CCs, like the 3.3 % difference seen in this study, have previously been linked with large differences in abundance (Nagel and Griffin 2001). In combination with its relatively high photosynthetic rates (Mozdzer and Zieman 2010; Mozdzer *et al.* 2013) and plastic nutrient use efficiency (Mozdzer and Megonigal 2012), introduced *Phragmites* has had an energetic advantage from its establishment to the present day that could have contributed to its invasiveness.

In contrast to our expectations, and unlike most performance metrics measured in introduced *Phragmites* under global change conditions (Holdredge *et al.* 2010; Mozdzer and Megonigal 2012; Eller *et al.* 2014), our plant-scale data suggest that advantages due to CC will diminish with rising atmospheric CO_2_ and nutrient proliferation. If efficient tissue construction and short payback time are particularly strong components of introduced *Phragmites* invasiveness, as global change intensifies, the competitive dynamics of these lineages may shift such that introduced *Phragmites* is less able to dominate ecosystems. However, other factors may allow for a continued competitive advantage by introduced *Phragmites*, especially if the increased investment in tissues improves their performance. Such factors include photosynthetic capacity (Mozdzer and Zieman 2010), salinity tolerance (Vasquez *et al.* 2005), production of litter that suppresses competing plants (Holdredge and Bertness 2011) and a propensity to outcross and generate greater numbers of seedlings at eutrophied sites (McCormick *et al.* 2010). In addition, like the processes that are selecting for genotypes well adapted to eutrophied conditions (McCormick *et al.* 2010), shifts in CO_2_ and N may similarly select for more efficient tissue construction in populations of introduced *Phragmites*.

## Conclusions

Leaf CCs alone do not provide an adequate representation of the energy required to produce biomass for *Phragmites*. Accounting for all major plant organs enabled us to identify key patterns in CCs, particularly belowground, that are likely associated with the invasive ability of the introduced lineage. In future studies attempting to address questions of plant carbon economy using CCs, we recommend that organs other than leaves be investigated, especially those belowground. In addition to gaining insight into invasion dynamics associated with rhizome and whole-plant CC patterns, these traits allowed us to identify responses to global change that are not well described in the literature. For instance, we observed greater root and stem CCs under elevated CO_2_ and greater leaf CC under high N. Given the critical nature of understanding plant responses to global change, scientists should use the full array of tools available.

## Sources of Funding

Funding for J.S.C. was provided by a Bucher-Jackson fellowship through Bryn Mawr College. T.J.M. was supported by a Smithsonian Institution fellowship at the time of the experiment. Additional financial support came from the National Science Foundation (award DEB-0950080), Maryland Sea Grant (award SA7528114-WW) and Bryn Mawr College.

## Contributions by the Authors

T.J.M. designed the experiment, T.J.M. and C.N.W. collected data, and J.S.C. analysed the data. All authors contributed to writing, led by J.S.C.

## Conflicts of Interest Statement

None declared.

## Supporting Information

The following Supporting Information is available in the online version of this article –

**Figure S1.** Construction costs vs. other functional traits by treatment, for native and introduced *Phragmites australis* (mean ± SE). See Table 2 for descriptions of traits.

Additional Information

## References

[PLU020C1] Amsberry L, Baker MA, Ewanchuk PJ, Bertness MD (2000). Clonal integration and the expansion of *Phragmites australis*. Ecological Applications.

[PLU020C2] Bart D, Hartman JM (2003). The role of large rhizome dispersal and low salinity windows in the establishment of common reed, *Phragmites australis*, in salt marshes: new links to human activities. Estuaries.

[PLU020C3] Baruch Z, Goldstein G (1999). Leaf construction cost, nutrient concentration, and net CO_2_ assimilation of native and invasive species in Hawaii. Oecologia.

[PLU020C4] Baxter I, Hosmani PS, Rus A, Lahner B, Borevitz JO, Muthukumar B, Mickelbart MV, Schreiber L, Franke RB, Salt DE (2009). Root suberin forms an extracellular barrier that affects water relations and mineral nutrition in Arabidopsis. PLoS Genetics.

[PLU020C5] Booker FL, Maier CA (2001). Atmospheric carbon dioxide, irrigation, and fertilization effects on phenolic and nitrogen concentrations in loblolly pine (*Pinus taeda*) needles. Tree Physiology.

[PLU020C6] Booker FL, Shafer SR, Wei CM, Horton SJ (2000). Carbon dioxide enrichment and nitrogen fertilization effects on cotton (*Gossypium hirsutum* L.) plant residue chemistry and decomposition. Plant and Soil.

[PLU020C7] Brix H, Sorrell BK, Orr PT (1992). Internal pressurization and convective gas-flow in some emergent fresh-water macrophytes. Limnology and Oceanography.

[PLU020C8] Chambers RM, Meyerson LA, Saltonstall K (1999). Expansion of *Phragmites australis* into tidal wetlands of North America. Aquatic Botany.

[PLU020C9] Curtis PS, Wang XZ (1998). A meta-analysis of elevated CO_2_ effects on woody plant mass, form, and physiology. Oecologia.

[PLU020C10] Deng X, Ye WH, Feng HL, Yang QH, Cao HL, Hui KY, Zhang Y (2004). Gas exchange characteristics of the invasive species *Mikania micrantha* and its indigenous congener *M. cordata* (Asteraceae) in South China. Botanical Bulletin of Academia Sinica.

[PLU020C11] Eller F, Lambertini C, Nguyen LX, Brix H (2014). Increased invasive potential of non-native *Phragmites australis*: elevated CO_2_ and temperature alleviate salinity effects on photosynthesis and growth. Global Change Biology.

[PLU020C12] Feng Y-L, Fu G-L, Zheng Y-L (2008). Specific leaf area relates to the differences in leaf construction cost, photosynthesis, nitrogen allocation, and use efficiencies between invasive and noninvasive alien congeners. Planta.

[PLU020C13] George K, Norby RJ, Hamilton JG, DeLucia EH (2003). Fine-root respiration in a loblolly pine and sweetgum forest growing in elevated CO_2_. New Phytologist.

[PLU020C14] Granéli W, Weisner SB, Sytsma M (1992). Rhizome dynamics and resource storage in *Phragmites australis*. Wetlands Ecology and Management.

[PLU020C15] Griffin K, Thomas R, Strain B (1993). Effects of nitrogen supply and elevated carbon dioxide on construction cost in leaves of *Pinus taeda* (L.) seedlings. Oecologia.

[PLU020C16] Hansen DL, Lambertini C, Jampeetong A, Brix H (2007). Clone-specific differences in *Phragmites australis*: effects of ploidy level and geographic origin. Aquatic Botany.

[PLU020C17] Hazelton ELG, Mozdzer TJ, Burdick DM, Kettenring KM, Whigham DF (2014). *Phragmites australis* management in the United States: 40 years of methods and outcomes. AoB PLANTS.

[PLU020C18] Holdredge C, Bertness MD (2011). Litter legacy increases the competitive advantage of invasive *Phragmites australis* in New England wetlands. Biological Invasions.

[PLU020C19] Holdredge C, Bertness MD, von Wettberg E, Silliman BR (2010). Nutrient enrichment enhances hidden differences in phenotype to drive a cryptic plant invasion. Oikos.

[PLU020C20] Hopkinson CS, Giblin AE (2008). Nitrogen dynamics of coastal salt marshes. Nitrogen in the marine environment.

[PLU020C21] Kettenring KM, de Blois S, Hauber DP (2012). Moving from a regional to a continental perspective of *Phragmites australis* invasion in North America. AoB PLANTS.

[PLU020C22] King RS, Deluca WV, Whigham DF, Marra PP (2007). Threshold effects of coastal urbanization on *Phragmites australis* (common reed) abundance and foliar nitrogen in Chesapeake Bay. Estuaries and Coasts.

[PLU020C23] Kraus TEC, Zasoski RJ, Dahlgren RA (2004). Fertility and pH effects on polyphenol and condensed tannin concentrations in foliage and roots. Plant and Soil.

[PLU020C24] Lambertini C, Sorrell BK, Riis T, Olesen B, Brix H (2012). Exploring the borders of European *Phragmites* within a cosmopolitan genus. AoB PLANTS.

[PLU020C25] Langley JA, Mozdzer TJ, Shepard KA, Hagerty SB, Megonigal JP (2013). Tidal marsh plant responses to elevated CO_2_, nitrogen fertilization, and sea level rise. Global Change Biology.

[PLU020C26] League MT, Colbert EP, Seliskar DM, Gallagher JL (2006). Rhizome growth dynamics of native and exotic haplotypes of *Phragmites australis* (common reed). Estuaries and Coasts.

[PLU020C27] Lei YB, Wang WB, Feng YL, Zheng YL, Gong HD (2012). Synergistic interactions of CO_2_ enrichment and nitrogen deposition promote growth and ecophysiological advantages of invading *Eupatorium adenophorum* in Southwest China. Planta.

[PLU020C28] Madhu M, Hatfield JL (2013). Dynamics of plant root growth under increased atmospheric carbon dioxide. Agronomy Journal.

[PLU020C29] Marks M, Lapin B, Randall J (1994). *Phragmites australis* (*P. communis*): threats, management, and monitoring. Natural Areas Journal.

[PLU020C30] McCormick MK, Kettenring KM, Baron HM, Whigham DF (2010). Spread of invasive *Phragmites australis* in estuaries with differing degrees of development: genetic patterns, Allee effects and interpretation. Journal of Ecology.

[PLU020C31] Meinshausen M, Smith SJ, Calvin K, Daniel JS, Kainuma MLT, Lamarque J-F, Matsumoto K, Montzka SA, Raper SCB, Riahi K, Thomson A, Velders GJM, van Vuuren DPP (2011). The RCP greenhouse gas concentrations and their extensions from 1765 to 2300. Climatic Change.

[PLU020C32] Mozdzer TJ, Megonigal JP (2012). Jack-and-Master trait responses to elevated CO_2_ and N: a comparison of native and introduced *Phragmites australis*. PLoS ONE.

[PLU020C33] Mozdzer TJ, Megonigal JP (2013). Increased methane emissions by an introduced *Phragmites australis* lineage under global change. Wetlands.

[PLU020C34] Mozdzer TJ, Zieman JC (2010). Ecophysiological differences between genetic lineages facilitate the invasion of non-native *Phragmites australis* in North American Atlantic coast wetlands. Journal of Ecology.

[PLU020C35] Mozdzer TJ, Zieman JC, McGlathery KJ (2010). Nitrogen uptake by native and invasive temperate coastal macrophytes: importance of dissolved organic nitrogen. Estuaries and Coasts.

[PLU020C36] Mozdzer TJ, Brisson J, Hazelton ELG (2013). Physiological ecology and functional traits of North American native and Eurasian introduced *Phragmites australis* lineages. AoB PLANTS.

[PLU020C37] Nagel JM, Griffin KL (2001). Construction cost and invasive potential: comparing *Lythrum salicaria* (Lythraceae) with co-occurring native species along pond banks. American Journal of Botany.

[PLU020C38] Nagel JM, Huxman TE, Griffin KL, Smith SD (2004). CO_2_ enrichment reduces the energetic cost of biomass construction in an invasive desert grass. Ecology.

[PLU020C39] Nagel JM, Wang X, Lewis JD, Fung HA, Tissue DT, Griffin KL (2005). Atmospheric CO_2_ enrichment alters energy assimilation, investment and allocation in *Xanthium strumarium*. New Phytologist.

[PLU020C40] Osunkoya OO, Daud SD, Wimmer FL (2008). Longevity, lignin content and construction cost of the assimilatory organs of *Nepenthes* species. Annals of Botany.

[PLU020C41] Osunkoya OO, Bayliss D, Panetta FD, Vivian-Smith G (2010). Leaf trait co-ordination in relation to construction cost, carbon gain and resource-use efficiency in exotic invasive and native woody vine species. Annals of Botany.

[PLU020C42] Park MG, Blossey B (2008). Importance of plant traits and herbivory for invasiveness of *Phragmites australis* (Poaceae). American Journal of Botany.

[PLU020C43] Poorter H, Van Berkel Y, Baxter R, Den Hertog J, Dijkstra P, Gifford RM, Griffin KL, Roumet C, Roy J, Wong SC (1997). The effect of elevated CO_2_ on the chemical composition and construction costs of leaves of 27 C3 species. Plant, Cell and Environment.

[PLU020C44] Poorter H, Pepin S, Rijkers T, de Jong Y, Evans JR, Korner C (2006). Construction costs, chemical composition and payback time of high- and low-irradiance leaves. Journal of Experimental Botany.

[PLU020C45] Poorter H, Niklas KJ, Reich PB, Oleksyn J, Poot P, Mommer L (2012). Biomass allocation to leaves, stems and roots: meta-analyses of interspecific variation and environmental control. New Phytologist.

[PLU020C46] Poorter L, Bongers F (2006). Leaf traits are good predictors of plant performance across 53 rain forest species. Ecology.

[PLU020C47] Rolletschek H, Hartzendorf T, Rolletschek A, Kohl JG (1999). Biometric variation in *Phragmites australis* affecting convective ventilation and amino acid metabolism. Aquatic Botany.

[PLU020C48] Saltonstall K (2002). Cryptic invasion by a non-native genotype of the common reed, *Phragmites australis*, into North America. Proceedings of the National Academy of Sciences of the USA.

[PLU020C49] Saltonstall K (2007). Comparison of morphological variation indicative of ploidy level in *Phragmites australis* (Poaceae) from eastern North America. Rhodora.

[PLU020C50] Saltonstall K, Stevenson JC (2007). The effect of nutrients on seedling growth of native and introduced *Phragmites australis*. Aquatic Botany.

[PLU020C51] Shen X-Y, Peng S-L, Chen B-M, Pang J-X, Chen L-Y, Xu H-M, Hou Y-P (2011). Do higher resource capture ability and utilization efficiency facilitate the successful invasion of native plants?. Biological Invasions.

[PLU020C52] Silliman BR, Bertness MD (2004). Shoreline development drives invasion of *Phragmites australis* and the loss of plant diversity on New England salt marshes. Conservation Biology.

[PLU020C53] Sokal RR, Rohlf FJ (1995). Biometry: the principles and practice of statistics in biological research.

[PLU020C54] Soukup A, Votrubova O, Cizkova H (2002). Development of anatomical structure of roots of *Phragmites australis*. New Phytologist.

[PLU020C55] Tulbure MG, Ghioca-Robrecht DM, Johnston CA, Whigham DF (2012). Inventory and ventilation efficiency of nonnative and native *Phragmites australis* (common reed) in tidal wetlands of the Chesapeake Bay. Estuaries and Coasts.

[PLU020C56] Vance C, Kirk T, Sherwood R (1980). Lignification as a mechanism of disease resistance. Annual Review of Phytopathology.

[PLU020C57] Vasquez EA, Glenn EP, Brown JJ, Guntenspergen GR, Nelson SG (2005). Salt tolerance underlies the cryptic invasion of North American salt marshes by an introduced haplotype of the common reed *Phragmites australis* (Poaceae). Marine Ecology Progress Series.

[PLU020C58] Vertregt N, Penning de Vries FWT (1987). A rapid method for determining the efficiency of biosynthesis of plant biomass. Journal of Theoretical Biology.

[PLU020C59] Vretare V, Weisner SEB (2000). Influence of pressurized ventilation on performance of an emergent macrophyte (*Phragmites australis*). Journal of Ecology.

[PLU020C60] Wright IJ, Reich PB, Westoby M, Ackerly DD, Baruch Z, Bongers F, Cavender-Bares J, Chapin T, Cornelissen JH, Diemer M, Flexas J, Garnier E, Groom PK, Gulias J, Hikosaka K, Lamont BB, Lee T, Lee W, Lusk C, Midgley JJ, Navas ML, Niinemets U, Oleksyn J, Osada N, Poorter H, Poot P, Prior L, Pyankov VI, Roumet C, Thomas SC, Tjoelker MG, Veneklaas EJ, Villar R (2004). The worldwide leaf economics spectrum. Nature.

[PLU020C61] Wullschleger SD, Norby RJ, Love JC, Runck C (1997). Energetic costs of tissue construction in yellow-poplar and white oak trees exposed to long-term CO_2_ enrichment. Annals of Botany.

