# Supplementary material for: Belowground advantages in construction cost facilitate a cryptic plant invasion
Source: AoB Plants. 2014 Apr 30;6:plu020. doi: 10.1093/aobpla/plu020 (PMC4060782; doi:10.1093/aobpla/plu020)
Supplement: Additional Information [file supp_6_plu020_index.html]

Belowground advantages in construction cost facilitate a cryptic plant invasion — Additional Information 

# Belowground advantages in construction cost facilitate a cryptic plant invasion

## Additional Information

Additional Information

**Files in this Data Supplement:**

- Additional Information - pptx file
