# Supplementary figures and images for: Belowground advantages in construction cost facilitate a cryptic plant invasion
Source: AoB Plants. 2014 Apr 30;6:plu020. doi: 10.1093/aobpla/plu020 (PMC4060782; doi:10.1093/aobpla/plu020)

## Slide 1
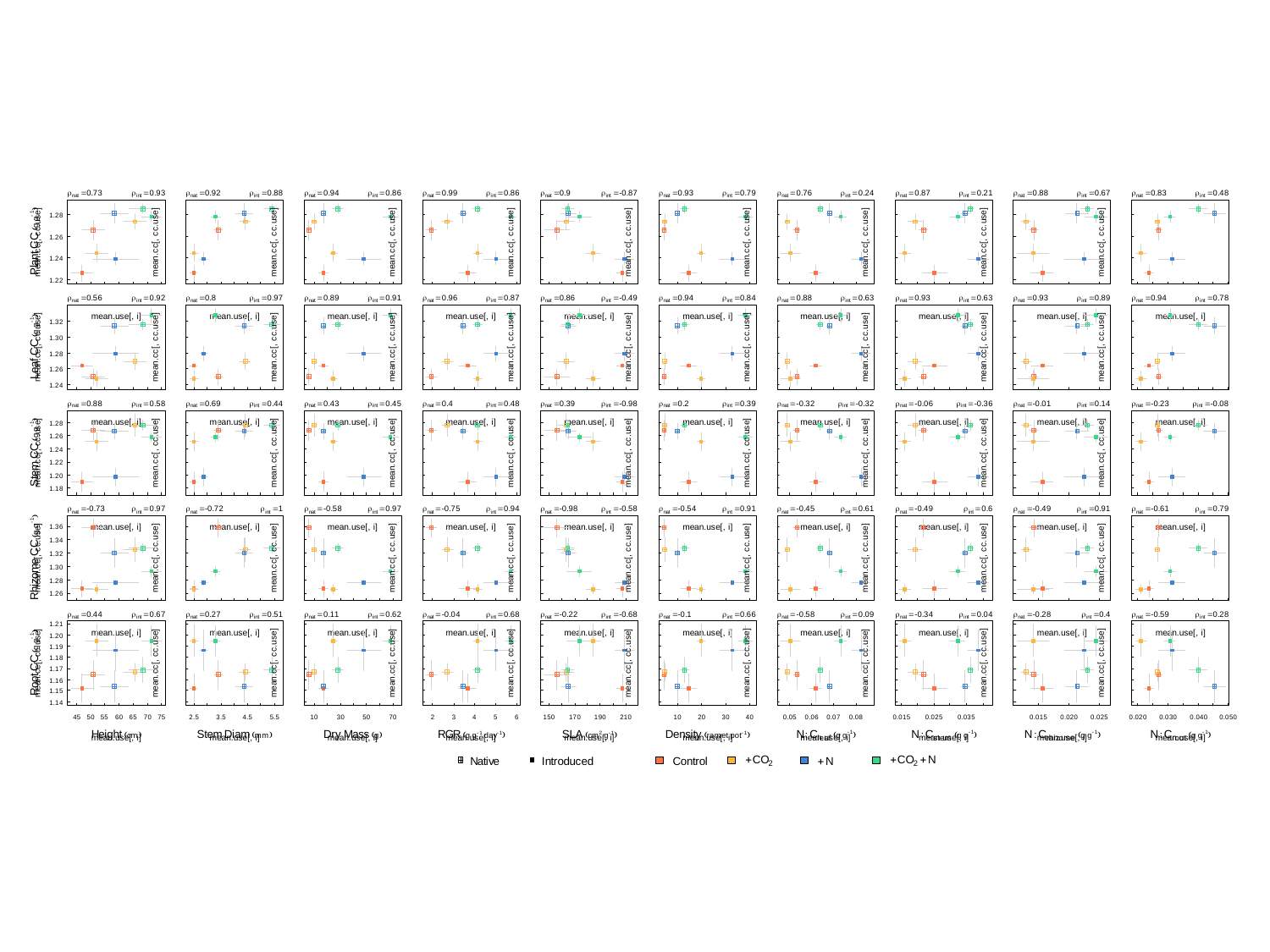

Supplement: Additional Information [file supp_plu020_plu020supp.pptx]
